# Genome-Wide Characterization and Expression Analysis of HD-ZIP Gene Family in *Dendrobium officinale*

**DOI:** 10.3389/fgene.2022.797014

**Published:** 2022-03-18

**Authors:** Qianyu Yang, Weibo Xiang, Zhihui Li, Yuxin Nian, Xiaoyun Fu, Guangzhu Zhou, Linbao Li, Jun Zhang, Guiyun Huang, Xiao Han, Lu Xu, Xiao Bai, Lei Liu, Di Wu

**Affiliations:** ^1^ College of Forestry, Shenyang Agricultural University, Shenyang, China; ^2^ Rare Plants Research Institute of Yangtze River, China Three Gorges Corporation, Yichang, China; ^3^ National Engineering Research Center of Eco-Environment Protection for Yangtze River Economic Belt, China Three Gorges Corporation, Beijing, China; ^4^ YANGTZE Eco-Environment Engineering Research Center, China Three Gorges Corporation, Beijing, China; ^5^ Natural Resources Affairs Service Center of Dalian, Dalian, China; ^6^ College of Horticulture, Hunan Agricultural University, Hunan Mid-Subtropical Quality Plant Breeding and Utilization Engineering Technology Research Center, Changsha, China; ^7^ State Key Laboratory of Tree Genetics and Breeding, Key Laboratory of Tree Breeding and Cultivation of the State Forestry Administration, Research Institute of Forestry, Chinese Academy of Forestry, Beijing, China

**Keywords:** HD-ZIP gene family, cold stress, *Dendrobium officinale*, expression profiles, transcription factor

## Abstract

The homeodomain-leucine zipper (HD-ZIP) gene family, as one of the plant-specific transcription factor families, plays an important role in regulating plant growth and development as well as in response to diverse stresses. Although it has been extensively characterized in many plants, the HD-ZIP family is not well-studied in *Dendrobium officinale*, a valuable ornamental and traditional Chinese medicinal herb. In this study, 37 HD-ZIP genes were identified in *Dendrobium officinale* (*Dohdzs*) through the *in silico* genome search method, and they were classified into four subfamilies based on phylogenetic analysis. Exon–intron structure and conserved protein domain analyses further supported the prediction with the same group sharing similar gene and protein structures. Furthermore, their expression patterns were investigated in nine various tissues and under cold stress based on RNA-seq datasets to obtain the tissue-specific and cold-responsive candidates. Finally, *Dohdz5*, *Dohdz9*, and *Dohdz12* were selected to validate their expression through qRT-PCR analysis, and they displayed significantly differential expression under sudden chilling stress, suggesting they might be the key candidates underlying cold stress response. These findings will contribute to better understanding of the regulatory roles of the HD-ZIP family playing in cold stress and also will provide the vital targets for further functional studies of HD-ZIP genes in *D. officinale*.

## 1 Introduction

The homeodomain-leucine zipper (HD-ZIP) gene family is one of the most important transcription factor families in plants involved in regulating growth, development, and in response to diverse abiotic and biotic stresses (Hu et al., 2016). It is a relatively evolutionarily conserved family that is only found in higher plants (Elhiti and Stasolla, 2009). To date, the HD-ZIP family has been extensively characterized in a large number of plant species, such as Arabidopsis (Henriksson et al., 2005), rice (Agalou et al., 2008), maize (Zhao et al., 2011), *Nicotiana tabacum* (Li et al., 2019a), and potato (Li et al., 2019b). Generally, HD-ZIP proteins possess a conserved homeodomain (HD), which is a specific DNA binding site at the C-terminal, and a conserved adjacent leucine zipper (LZ) motif that is responsible for protein dimerization (Ruberti et al., 1991). According to their sequence homology, DNA binding specificity, and physiological functioning, HD-ZIP genes were further divided into four subfamilies, HD-ZIP I–IV (Ariel et al., 2007). HD-ZIP I and II families contained the conserved HD and LZ domains, which could bind to the similar sequence CAAT-N-ATTG (Hu et al., 2012). At the same time, members of the HD-ZIP II subfamily also contain a CPSCE (Cys–Pro–Ser–Cys–Glu) motif consisting of five conserved amino acids after the C-terminus of the LZ domain. Furthermore, HD-ZIP III and IV families possessed the START (steroidogenic acute regulatory protein-related lipid transfer) and SAD (START-associated domain) domains with putative lipid-binding capability, except for HD and LZ domains, while HD-ZIP III also had a specific MEKHLA domain located in the C-terminus, which did not exist in HD-ZIP IV (Ponting and Aravind, 1999). In addition, previous studies have found that HD-ZIP proteins showed different subcellular localization, including nuclei, mitochondria, and cytoplasm, suggesting that HD-ZIP genes could participate in diverse biological processes.

It has been extensively demonstrated that the HD-ZIP genes play an important role in regulating plant growth and environmental responses (Sessa et al., 2018; Cristina et al., 1996). HD-ZIP I proteins were found to be involved in regulating plant development (Mao et al., 2016; Gong et al., 2019). Kovalchuk et al. (2016) demonstrated that the *TaHDZip I-2* gene regulated wheat flowering and spike development, and it improved the frost tolerance of the transgenic barley lines. LcHB2 and LcHB3, belonging to the HD-ZIP I subfamily, are involved in fruitlet abscission by promoting transcription of the genes related to biosynthesis of ethylene and ABA in litchi (Li et al., 2019c). HD-ZIP II proteins mainly regulated hormone signaling, participating in embryonic apical development and response to light and abiotic stresses. Chen et al. (2019a) reported that a small grain mutant dwarf 2 encoded an HD-ZIP II transcription factor to control grain development by modulating gibberellin biosynthesis in rice. Gu et al. (2019) found that an HD-ZIP II transcription factor, *PpHB.G7*, could mediate ethylene biosynthesis during fruit ripening in peach. GmZPR3d was found to interact with GmHD-ZIP III proteins to regulate root and nodule vascular development in soybean (Damodaran et al., 2019). Wang et al. (2020) revealed that an HD-ZIP IV transcription factor NtHDG2 regulated flavonol biosynthesis to promote trichome development in *Nicotiana tabacum*. The HD-ZIP IV gene, *PDF2*, played a vital role in the epidermis cells to regulate normal development of floral organs in Arabidopsis (Kamata et al., 2013). In maize, *OCL4* inhibited trichome development and influenced another cell division and differentiation (Vernoud et al., 2009). Cold stress is one of the most destructive environmental factors limiting plant growth and development in higher latitude regions, which will directly impair cell fluidity to damage the photosynthetic process and other metabolic activities (He et al., 2021). Some studies have demonstrated that the HD-ZIP family played an important role in regulating cold stress resistance (Sharif et al., 2021). It is reported that transgenic Arabidopsis with overexpression of *AtHB13* displayed stronger cold stress tolerance than wild type (WT) by maintaining cellular stability under low-temperature conditions (Gong et al., 2019). Overexpression of the sunflower HD-ZIP I family gene *HaHB1* in sunflower and soybean could improve resistance to cold stress by regulating the expression of some cell membrane–related proteins (Cabello et al., 2012a). Similar to TaHDZipI-2, overexpressing TaHDZipI-5 could improve cold tolerance to ensure normal flowering when suffering from freezing stress at the reproductive stage (Yang et al., 2018a).

*Dendrobium officinale* is a herb with high medicinal and ornamental significance, containing abundant polysaccharides, dendrobium alkaloids, flavonoids, and other bioactive substances (Yuan et al., 2020). Due to its rareness and valuable medicinal function, *D. officinale* is referred to as a “medicinal giant panda,” providing an indispensable plant resource for traditional Chinese medicine and high-end healthcare product. Therefore, it has high research value and large utilization prospects. Abiotic stress is one of the most destructive factors limiting the production of *D. officinale*, which not only adversely affected the growth and development of *D. officinale* but also damaged its medicinal and ornamental value, especially cold stress (Chen et al., 2017). Thus, it is crucial to identify and use an elite gene resource to improve the cold tolerance of *D. officinale*. Although increasing studies have found that several HD-ZIP genes regulated cold tolerance in plants (Kovalchuk et al., 2012; Yang et al., 2018b), their importance of them in *D. officinale* has not yet been studied. Here, we systematically characterized the HD-ZIP family in *D. officinale* at the genome scale, and then their structure, phylogeny, conserved motifs, and expression profiles were also comprehensively investigated. The results will provide vital genetic resources to improve the cold stress tolerance and will help reveal the molecular mechanism of development and stress response in *D. officinale.*

## 2 Materials and Methods

### 2.1 Identification of HD-ZIP Genes in *Dendrobium officinale*

To identify HD-ZIP genes in *D. officinale*, all protein sequences annotated in the *D. officinale* genome were retrieved from the Herbal Medicine Omics Database (http://202.203.187.112/herbalplant/) to be used as the local protein database. HD-ZIP genes in Arabidopsis and rice were downloaded from the TAIR (https://www.arabidopsis.org/) and Rice database (http://rice.plantbiology.msu.edu/) to perform a BLASTP search against the local protein database with the threshold of E-value < 1e^−5^. The PFAM profile (PF00046) was downloaded from the PFAM database (https://pfam.xfam.org/) and used as the query to search against the local protein database using HMMER 3.0, with the threshold of E-value < 1e^−5^. The results of the HMMER and BLASTP search were integrated, and the redundancies were manually removed. The putative HD-ZIP genes were further checked by the NCBI conserved domain database (CDD) and Simple Modular Architecture Research Tool (SMART) to identify the conserved protein domain. Those that contained the complete HD and LZ domains remained as candidate HD-ZIP genes in *D. officinale* (Dohdzs).

### 2.2 Characteristics of HD-ZIP Genes in *Dendrobium officinale*

These predicted Dohdz proteins were submitted to the EXPASY database (https://web.expasy.org/) to calculate the isoelectric point (pI) and molecular weight (MW). Subcellular localization of them was predicted by the TargetP website tool (http://www.cbs.dtu.dk/services/TargetP/). For gene structure analysis, the corresponding genomic sequences of the identified Dohdzs were obtained from the reference sequences of *D. officinale* (DDBJ/EMBL/GenBank accession code: JSDN00000000). Genomic and CDS sequences were used for drawing gene structure schematic diagrams using the Gene Structure Display Server (http://gsds.cbi.pku.edu.cn/index.php).

### 2.3 Conserved Motif, Phylogenetic, and Cis-Element Analysis

Each Dohdz protein was submitted to the SMART database (http://smart.embl-heidelberg.de/) to predict the conserved domains. Then, protein sequences of HD-ZIP genes in *D. officinale,* Arabidopsis, and rice were aligned by the ClutsalX1.83 tool and then used to build the phylogenetic tree. The ProtTest tool was first used to predict the best evolution model, and JTT + G + I + F was used as the best evolution model to build the evolution tree using RAxML software with 1000 bootstraps, and the phylogenetic tree was visualized using Figtree software. The upstream 2,000 bp region of each Dohdz gene was extracted and submitted to the PlantCARE database (http://bioinformatics.psb.ugent.be/webtools/plantcare/html/) to predict *cis*-elements.

### 2.4 Expression Analysis of Dohdz Genes Using RNA-Seq Datasets

RNA-Seq datasets of nine tissues, including the column, sepal, the white part of the root, green root tip, stem, leaf, lip, and flower buds, were downloaded from the NCBI sequence read archive (SRA) database with Accession No. PRJNA348403, and the RNA-seq data under cold stress were also downloaded from the SRA database with Accession No. PRJNA314400, which was then used to detect the expression profiles of the identified Dohdzs to identify tissue-specific or stress-responsive candidates. A trimmomatic tool was used to remove the sequencing adapters and low-quality reads to obtain clean reads. Then, the filtered reads were aligned to the reference genome by the Hisat2 tool. In addition, TPM was calculated and used to normalize the gene expression data. After the tissue expression data are transformed by zscore, it is displayed on the iTOL online tool together with the motif information. Then, the Tau value was calculated by referring to the method described by Yanai et al. (2005). Tau value varies from 0 to 1, where 0 means broad expression, while 1 means specific expression. The differential expressed gene was investigated using the DESeq2 tool with FDR <0.05 and |log2foldchange| > 1 serving as cutoffs.

To get some insights into the interaction of these identified Dohdz genes with other genes in *D. officinale,* the coexpression network was constructed using the WGCNA tools based on the expression data from abovementioned RNA-seq analysis, and then the network or modules that Dohdz genes were involved in were selected. The regulatory network of Dohdzs and other genes was visualized using Cytoscape v3.8.0, and the Dohdz genes were highlighted.

### 2.5 Plant Materials and Cold Stress Treatments

The seeds in the capsule were subsequently sown into culture bottles containing 120 ml of 1/2 MS medium (0.5 g/L NAA, 7 g/Lagar, and 30 g/L sucrose; pH 5.8–6.0) and further incubated in a culture room (12 h of daylight, 60 mmol/m^2^/s, 20 ± 1°C) for 30 days. In addition, the seedlings of *D. officinale* were used for cold stress treatment at 0°C in a growth chamber (40 μmol/m^2^/s, a 12-h photoperiod, and 60% relative humidity). Other seedlings were grown in a 20°C chamber with parallel growth conditions as control treatment. To detect the expression patterns of Dohdzs, leaves were collected after 4, 12, 16, 20, and 24 h treatment under control and cold stress conditions for RNA extraction. Six plants were pooled as one biological replicate and for each experiment, and three biological replicates were adopted.

### 2.6 QRT-PCR Analysis

Total RNA was extracted from the obtained samples using TRIzol reagent (Life Technologies, USA). Two ug RNA was used to synthesize cDNA using the Geneseed® II First Strand cDNA Synthesis Kit (Geneseed, China) according to the manufacturer’s protocol. A qRT-PCR experiment was performed using ABI 7500 instrument (ABI7500, ABI, Foster City, CA, USA) with Geneseed® qPCR SYBR® Green Master Mix (Geneseed, China) and the final RT-qPCR reaction mixture of 20 μl volume, including 10 μl of Geneseed® qPCR SYBR® Green Master Mix, 0.5 μl of each primer (10 μM), 0.4 μl 50x ROX Reference Dye 2, 2 μl of the cDNA template, and 7.6 μl of RNase free H_2_O. Thermal cycling parameters for the amplification are as follows: 95°C for 5 min, followed by 40 cycles at 95°C for 10 s, and 60°C for 34 s. The glyceraldehyde-3-phosphate dehydrogenase gene in *D. officinale* (*DoGAPDH*) (Accession No. KX524087.1) was used as the internal reference gene. Relative expression levels of the targeted genes were calculated by 2^−ΔΔCT^ methods. The primers used in this study are listed in Supplementary Table S1.

### 2.7 Statistical Analysis

All the data from more than three biological replicates were analyzed using SPSS 21.0 (SPSS, Inc., Chicago, IL, USA) software. Quantitative data were presented as mean ± SD. The significance of differences between control and cold stress treatment was assessed by the paired t-test. Significant differences were finally defined as *p* < 0.05.

## 3 Results

### 3.1 Identification of HD-ZIP Genes in *Dendrobium officinale*

To globally obtain the HD-ZIP genes in *Dendrobium officinale*, candidate HD-ZIP genes were explored through a genome-wide search against the reference genome of *Dendrobium officinale* using HMMSearch (PF00046) and BLASTP (e-value <= e^−5^) methods. After removing redundant sequences and confirming the presence of both HD and LZ domains, 37 putative HD-ZIP genes (designated as *Dohdz1-37*) were obtained. Sequence analysis showed that these HD-ZIP genes varied from 423 (*Dohdz1*) to 2703 (*Dohdz18*) bp in length, and their exon numbers varied from 1 (*Dohdz4*) to 28 (*Dohdz23*). Their molecular weight ranged from 16.3 (*Dohdz1*) to 99.5 (*Dohdz18*) kDa, and the pI value ranged from 4.72 (*Dohdz32*) to 9.54 (*Dohdz4*). Subcellular localization analysis indicated that most of the *Dohdz*s were located in the nucleus, followed by chloroplast, mitochondria, and another cytoplasm, suggesting that HD-ZIP genes might play diverse roles in different biological processes in *D. officinale* (Table 1)*.*

**TABLE 1:** Basic information of the HD-ZIP genes identified in *D. officinale*.

Gene ID	Gene name	Type	Scaffold	CDS size (bp)	pI	Mw (kDa)	Subcellular location
Dendrobium_GLEAN_10136284	Dohdz1	type II	scaffold180	423	8.58	16.33798	Nuclear
Dendrobium_GLEAN_10127826	Dohdz2	type II	scaffold441	504	9.41	19.0526	Nuclear
Dendrobium_GLEAN_10089955	Dohdz3	type II	scaffold2522	1341	9.33	50.0062	Nuclear
Dendrobium_GLEAN_10089647	Dohdz4	type I	scaffold2541	705	9.54	26.2	Chloroplast
Dendrobium_GLEAN_10088206	Dohdz5	type I	scaffold2667	1038	9.14	39.7	Nuclear
Dendrobium_GLEAN_10087455	Dohdz6	type I	scaffold2693	1536	5.26	56.7	Extracellular
Dendrobium_GLEAN_10081987	Dohdz7	type I	scaffold3193	876	4.77	32.5	Nuclear
Dendrobium_GLEAN_10078433	Dohdz8	type II	scaffold3482	783	8.94	29.1	Nuclear
Dendrobium_GLEAN_10076418	Dohdz9	type I	scaffold3660	753	6.55	28.3	Chloroplast
Dendrobium_GLEAN_10074897	Dohdz10	type IV	scaffold3805	2118	5.85	78.5	Nuclear
Dendrobium_GLEAN_10070645	Dohdz11	type II	scaffold4236	795	9.16	29.4	Chloroplast
Dendrobium_GLEAN_10070394	Dohdz12	type II	scaffold4260	879	6.87	32.6	Nuclear
Dendrobium_GLEAN_10068306	Dohdz13	type II	scaffold4451	504	9.41	19.1	Nuclear
Dendrobium_GLEAN_10064494	Dohdz14	type I	scaffold4837	1011	5.21	37.8	Nuclear
Dendrobium_GLEAN_10064751	Dohdz15	type IV	scaffold4929	1599	5.7	57.4	Chloroplast
Dendrobium_GLEAN_10063217	Dohdz16	type I	scaffold5026	792	9.42	30.0	Nuclear
Dendrobium_GLEAN_10057787	Dohdz17	type II	scaffold5725	822	6.56	30.2	Nuclear
Dendrobium_GLEAN_10055614	Dohdz18	type III	scaffold6024	2703	6.25	99.5	Nuclear
Dendrobium_GLEAN_10051785	Dohdz19	type II	scaffold6549	504	9.47	18.3	Mitochondrion
Dendrobium_GLEAN_10047255	Dohdz20	type I	scaffold7245	660	4.56	23.4	Nuclear
Dendrobium_GLEAN_10045285	Dohdz21	type IV	scaffold7565	2160	5.52	78.7	Nuclear
Dendrobium_GLEAN_10044654	Dohdz22	type II	scaffold7738	831	5.43	31.0	Nuclear
Dendrobium_GLEAN_10044395	Dohdz23	type III	scaffold7796	3900	5.89	141.3	Nuclear
Dendrobium_GLEAN_10042663	Dohdz24	type IV	scaffold8039	2445	5.87	88.3	Nuclear
Dendrobium_GLEAN_10040440	Dohdz25	type IV	scaffold8454	2379	5.6	85.5	Nuclear
Dendrobium_GLEAN_10036995	Dohdz26	type IV	scaffold9340	2148	6.32	79.6	Nuclear
Dendrobium_GLEAN_10035608	Dohdz27	type I	scaffold9470	1155	9.13	44.3	Extracellular
Dendrobium_GLEAN_10030331	Dohdz28	type II	scaffold10849	705	9.3	2.6	Nuclear
Dendrobium_GLEAN_10028660	Dohdz29	type I	scaffold11248	660	4.79	24.4	Nuclear
Dendrobium_GLEAN_10028662	Dohdz30	type I	scaffold11248	585	4.84	21.4	Nuclear
Dendrobium_GLEAN_10027163	Dohdz31	type IV	scaffold11680	2415	5.5	86.8	Nuclear
PEQU_11247-D1	Dohdz32	type I	scaffold12132	771	4.72	29.4	Nuclear
Dendrobium_GLEAN_10021094	Dohdz33	type I	scaffold14011	627	8.69	23.9	Chloroplast
PEQU_10044-D5	Dohdz34	type I	scaffold14011	624	6.69	24.2	Nuclear
Dendrobium_GLEAN_10019904	Dohdz35	type II	scaffold14566	621	9.04	24.2	Nuclear
Dendrobium_GLEAN_10011239	Dohdz36	type I	scaffold21401	717	6.87	27.7	Nuclear
Dendrobium_GLEAN_10010656	Dohdz37	type I	scaffold22145	750	5.17	27.4	Nuclear
Average	—	—	—	1184.8	7.01	43.10	Nuclear

### 3.2 Phylogenetic and Conserved Domain Analysis of HD-ZIP Genes in *Dendrobium officinale*

**FIGURE 1:**
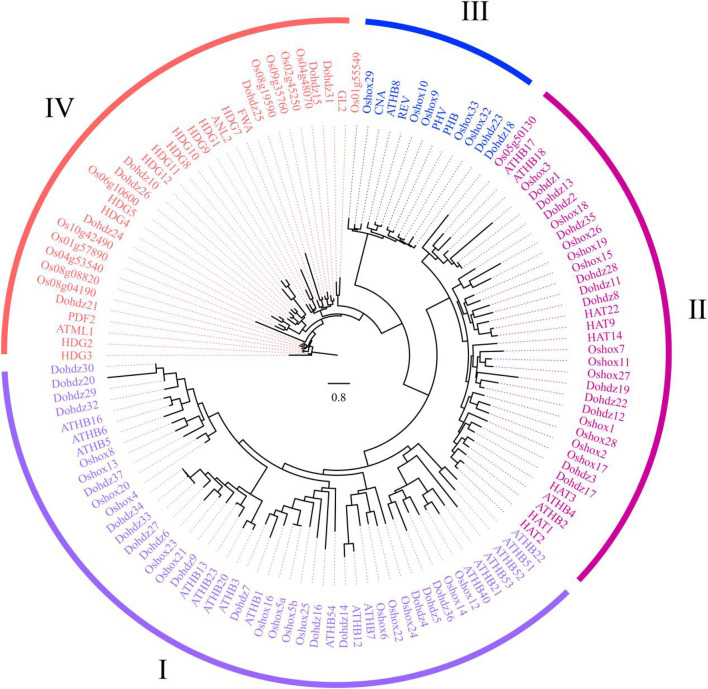
Phylogenetic analysis of HD-ZIP proteins in *D, officinale* (37), Arabidopsis (48), and rice (39) based on the Maximum Likelihood (ML) method using RAxML software.

To investigate the phylogenetic relationships, 37 HD-ZIP genes in *D. officinale*, together with 48 *Arabidopsis* and 39 rice HD-ZIP genes were selected for constructing a phylogenetic tree (Table 2). Based on the classification criteria in *Arabidopsis* and rice, these *Dohdz* proteins clustered into four groups. There are 12, 16, 2, and 7 *Dohdz* genes in I to IV groups, respectively. Further analysis showed that every group contained the HD-ZIP genes from *D. officinale*, *Arabidopsis,* and rice, indicating that there no obvious gene gain or loss event occurring between them, and the genetic divergence is earlier than that of monocotyledonous and dicotyledonous plants (Figure 1).

**TABLE 2:** Comparison of the number of HD-Zip genes in D. officinale wheat with seven other species.

Species	Group I	Group II	Group III	Group IV	Total
Arabidopsis	17	10	5	16	48
Soybean	28	27	12	19	86
Eucalyptus grandis	15	12	4	9	40
Dendrobium officinale	12	16	2	7	37
Rice	14	12	5	8	39
Maize	17	18	5	15	55
Brachypodium	16	19	6	18	59
Wheat	31	32	14	36	113

**FIGURE 2:**
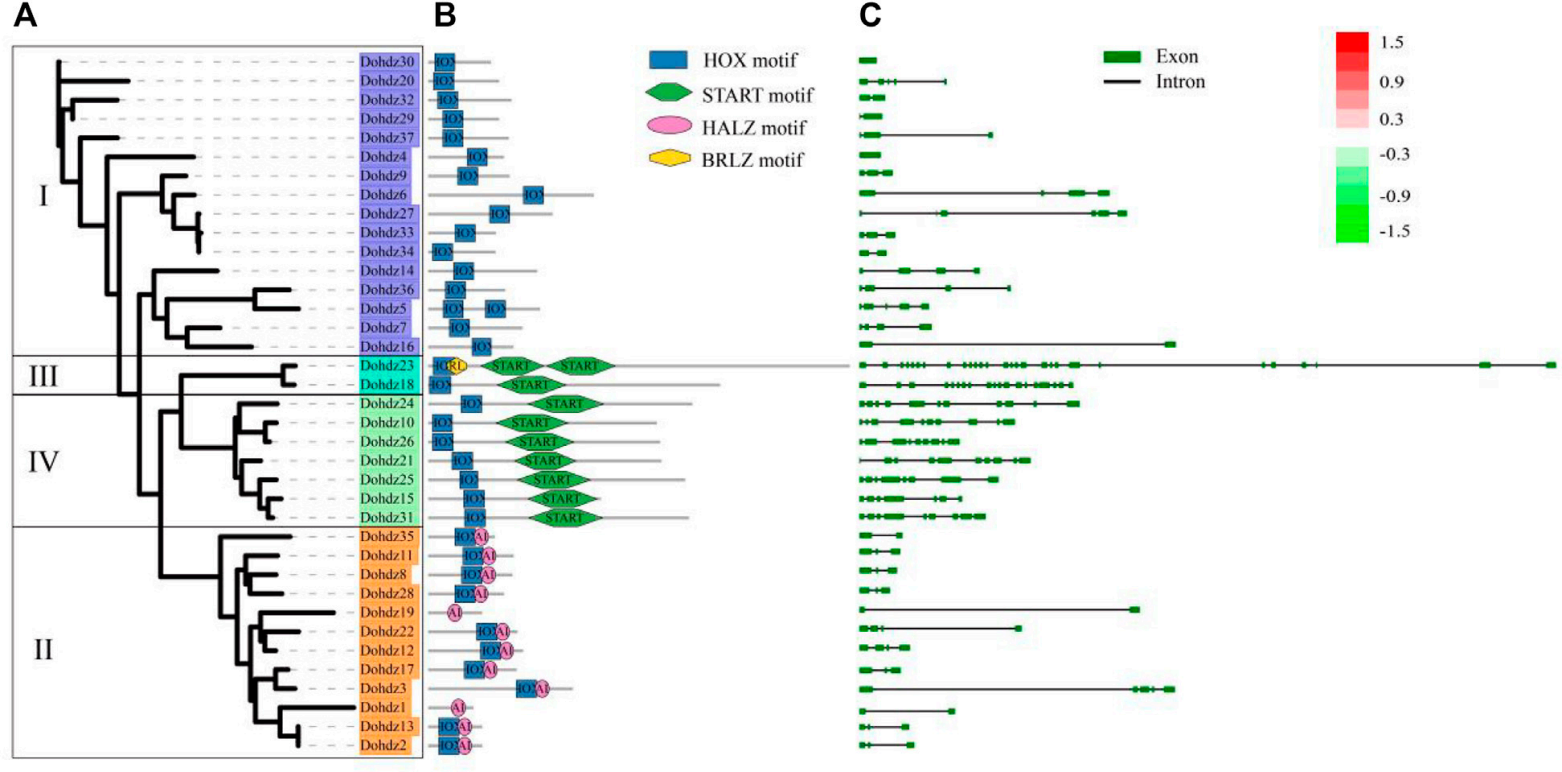
Phylogenetic relationships **(A)**, conserved motif compositions **(B),** and exon–intron structure **(C)** of these 37 HD-ZIP genes in *D. officinale*. The phylogenetic tree was constructed based on the full-length protein sequences using Figtree software; the conserved motifs were predicted using the SMART database. Each motif is represented by a different colored box; Gene exons and introns were indicated by boxes and lines.

Furthermore, the conserved functional domain in these *Dohdz* proteins was investigated. The results showed that four highly conserved functional domains were found, including HOX motif, SMART motif, HALZ motif, and BRLZ motif (Figure 2B). All *Dohdz* proteins contained the HOX motif, and group II had only the HOX domain. Group I and IV also contained BRLZ and START motifs, respectively. The conserved domain organization was completely consistent with the subfamily classification criteria. It is no accident that the members in the same subfamily have similar conserved motif compositions, suggesting their similar biological function.

Additionally, the exon–intron structures of these *Dohdzs* were also analyzed. The results showed that they had variable intron numbers ranging from 0 to 27 (Figure 2C), of which *Dohdz4* and *Dohdz30* had no intron, while *Dohdz18* and *Dohdz23* contained 18 and 27 introns, respectively. The members in the same subfamily have similar intron numbers and exon–intron structures. Subfamilies I and II have less introns with the number no more than 5. Conversely, Subfamilies III and IV have significantly rich intron numbers and more sophisticated gene structure.

### 3.3 *Cis*-Element Analysis of HD-ZIP Genes in *Dendrobium officinale*

**FIGURE 3:**
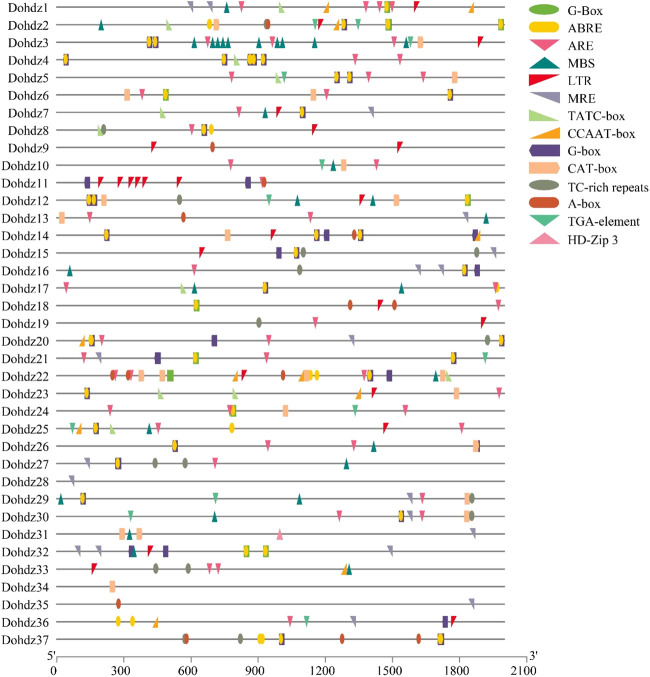
*Cis*-elements found in the promoter regions of these 37 HD-ZIP genes in *D. officinale.*

*Cis*-element is one kind of the most important regulatory factors in controlling gene transcription and expression, which could also provide some clues about gene functions (Agalou et al., 2008). Through prediction, 14 unique *cis*-element motifs were found in these 37 Dohdz genes belonging to different functional groups associated with plant development and stress response (Figure 3) (Supplementary Table S2). The phytohormone-responsive elements were widely found in the promoters of these Dohdz genes, including 62 ARBE elements (*cis*-acting element involved in the abscisic acid responsiveness), 13 TGA-element (auxin-responsive element), and 11 TATC-box elements (*cis*-acting element involved in gibberellin responsiveness), suggesting that these genes could be involved in hormone signaling transduction to regulate growth and stress response in *D. officinale.* Furthermore, 12 *Dohdz* genes were found to contain TC-rich repeat element (*cis*-acting element involved in defense and stress responsiveness), 18 Dohdz genes contained MBS element (MYB binding site involved in drought inducibility), and 19 Dohdz genes contained LTR element (*cis*-acting element involved in low-temperature responsiveness), suggesting that these *Dohdzs* might play crucial roles in regulating drought and cold stress response. Among them, *Dohdz11* had six LTR elements, *Dohdz9* have two LTR elements, and the others had one LRT element.

### 3.4 Expression Profile of Dohdzs in Different Tissues

**FIGURE 4:**
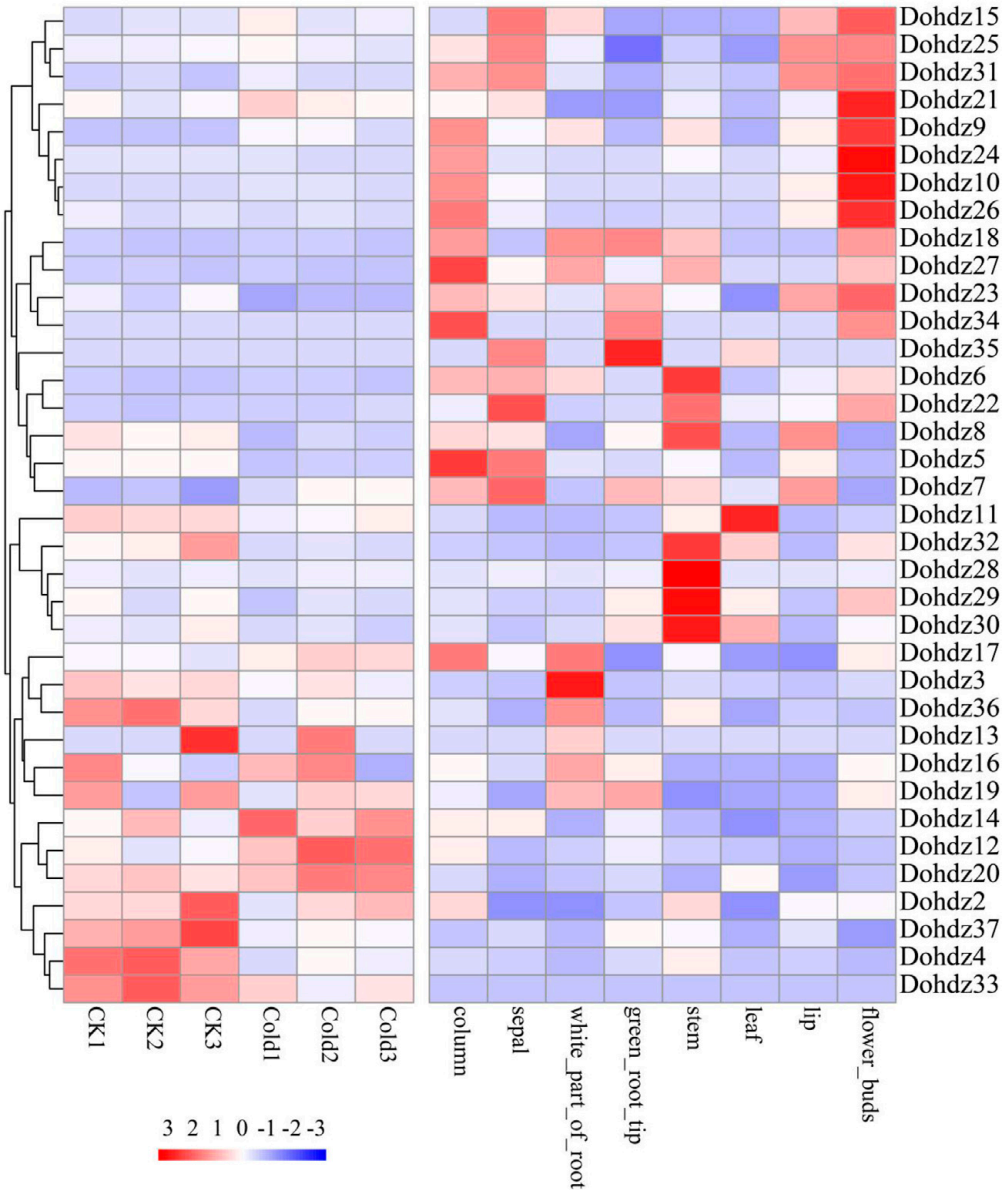
Expression patterns of these 37 HD-ZIP genes in different tissues and under cold stress.

Generally, different members of gene families exhibit disparities in abundance in different tissues (He et al., 2017). To get some insights into the putative biological functions of these *Dohdzs*, their temporal and spatial expression profile was comprehensively analyzed using the public RNA-seq data (PRJNA348403) (Figure 4). The results showed that subfamily I expressed in different tissues; subfamily II mainly expressed in the stem, column, and sepal; and subfamilies III and IV were tissue-specific and preferred to express in flower buds (Supplementary Table S3). These results indicated that different subfamilies of HD-ZIP genes played differential roles in regulating tissue development. Furthermore, the tissue-specific candidates were identified. *Dohdz 27* was found to be column-specific; *Dohdz36* and *Dohdz3* were specific in the white part of the root. *Dohdz37* showed specifically high expression in green root tip, and *Dohdz28* showed specifically high expression in the green root stem. The tissue-specific genes could contribute to different tissue morphogenesis. Interestingly, most of the tissue-specific *Dohdz* gene contained the G-Box element (cis-acting regulatory element involved in light responsiveness) and CAT-box element (cis-acting regulatory element related to meristem expression), indicating that the *cis*-elements could regulate the gene’s specific expression. Additionally, seven *Dohdz* genes did not express in any tissues, including *Dohdz1*,*2*,*13*,*16*,*33*,*34*, and *35*, which might not function on tissue development. In addition, we found that the expression of HD-ZIP genes showed increased tissue specificity compared to that of the whole genes in *D. officinale* (Supplementary Figure S1), suggesting the potential roles played by the HD-ZIP family in plant growth and development.

### 3.5 Expression Analysis of Dohdzs Under Cold Stress

To obtain the candidates related to cold tolerance, we further investigated the expression patterns of these identified Dohdzs under cold stress (Figure 4). The results found that 34 Dohdzs were expressed under cold stress conditions, of which 14 displayed significantly differential expression compared to control condition, with nine downregulated and five upregulated genes. The expression level of *Dohdz5* was 10-fold lower in cold stress than that of the control, while *Dohdz9* and *Dohdz12* were significantly upregulated. Further analysis showed that these DEGs belonged to different subfamilies, indicating that different subfamilies, rather than a specific subfamily of HD-ZIP genes, were involved in cold stress response and tolerance in *D. officinale*.

**FIGURE 5:**
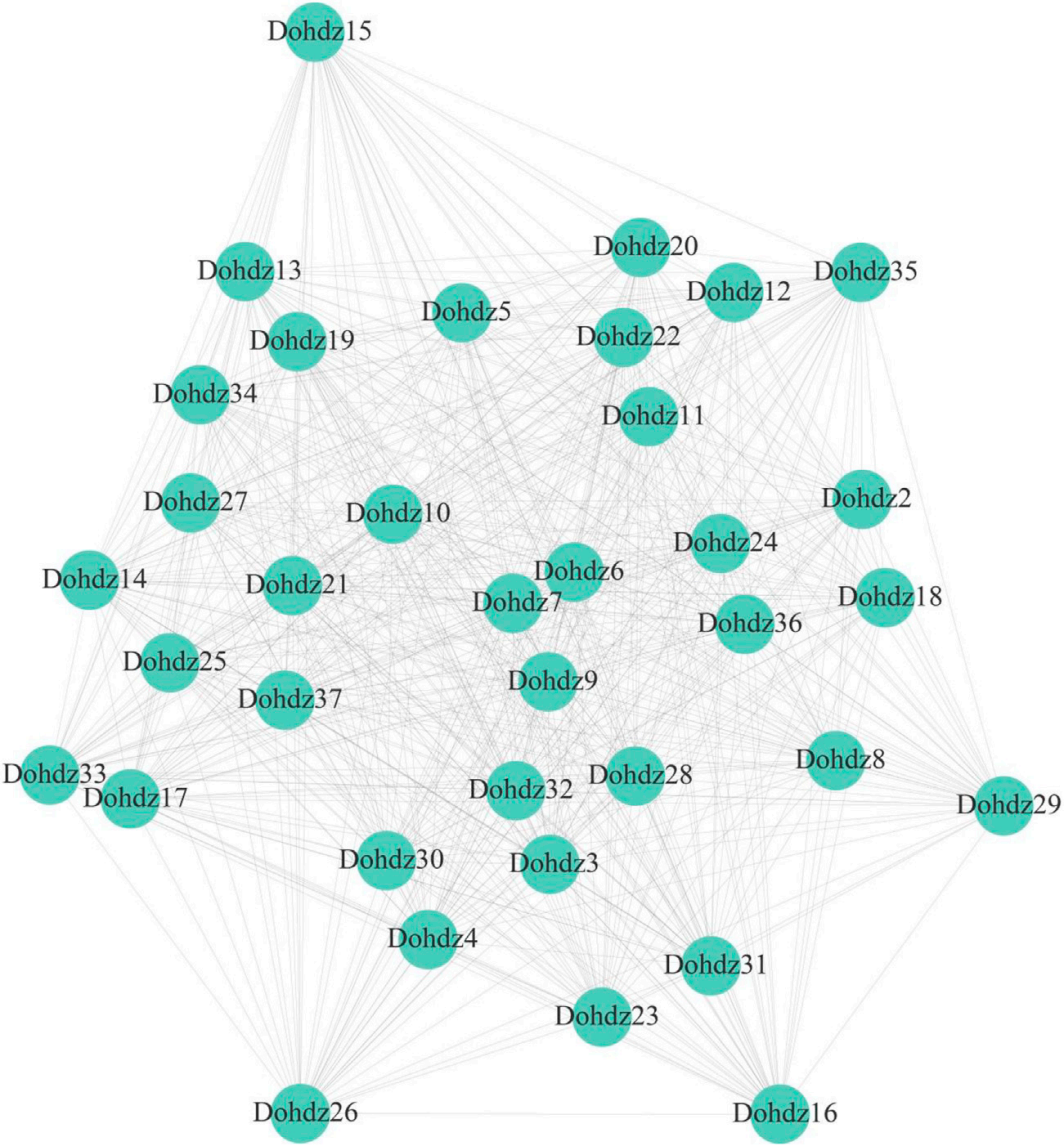
Coexpression network of Dohdz genes involved in regulating other genes in *D. officinale*.

Furthermore, the interaction relationship among *Dohdzs* and other genes in *D. officinale* was investigated through WGCNA analysis based on the expression levels in different tissues and under cold stress (Figure 5). A total of 13 coexpression modules were obtained, and these *Dohdzs* interacted with each other and also interacted with other functional genes to form a sophisticated network. Among them, *Dohdz37* was found to interact with other *34 Dohdzs*, which could be the hub gene in the Dohdz-mediated regulatory network. *Dohdz21* was found to interact with other 10 HD-ZIP genes and six functional genes, which is the orthologous gene of *PDF2* in Arabidopsis, suggesting that *Dohdz21* might interact with other genes to form the network to regulate floral organ development in *D. officinale.* Additionally, the cold-related *Dohdz5*, *Dohdz9*, and *Dohdz12* were also found to interact with many other genes. These modules could be involved in the regulatory network associated with the response and tolerance to cold stress. In conclusion, the coexpression network analysis of Dohdz genes contributed to better understanding their regulatory roles and function.

### 3.6 Validation of the Expression of *Dohdzs* Under Cold Stress by qRT-PCR Analysis

**FIGURE 6:**
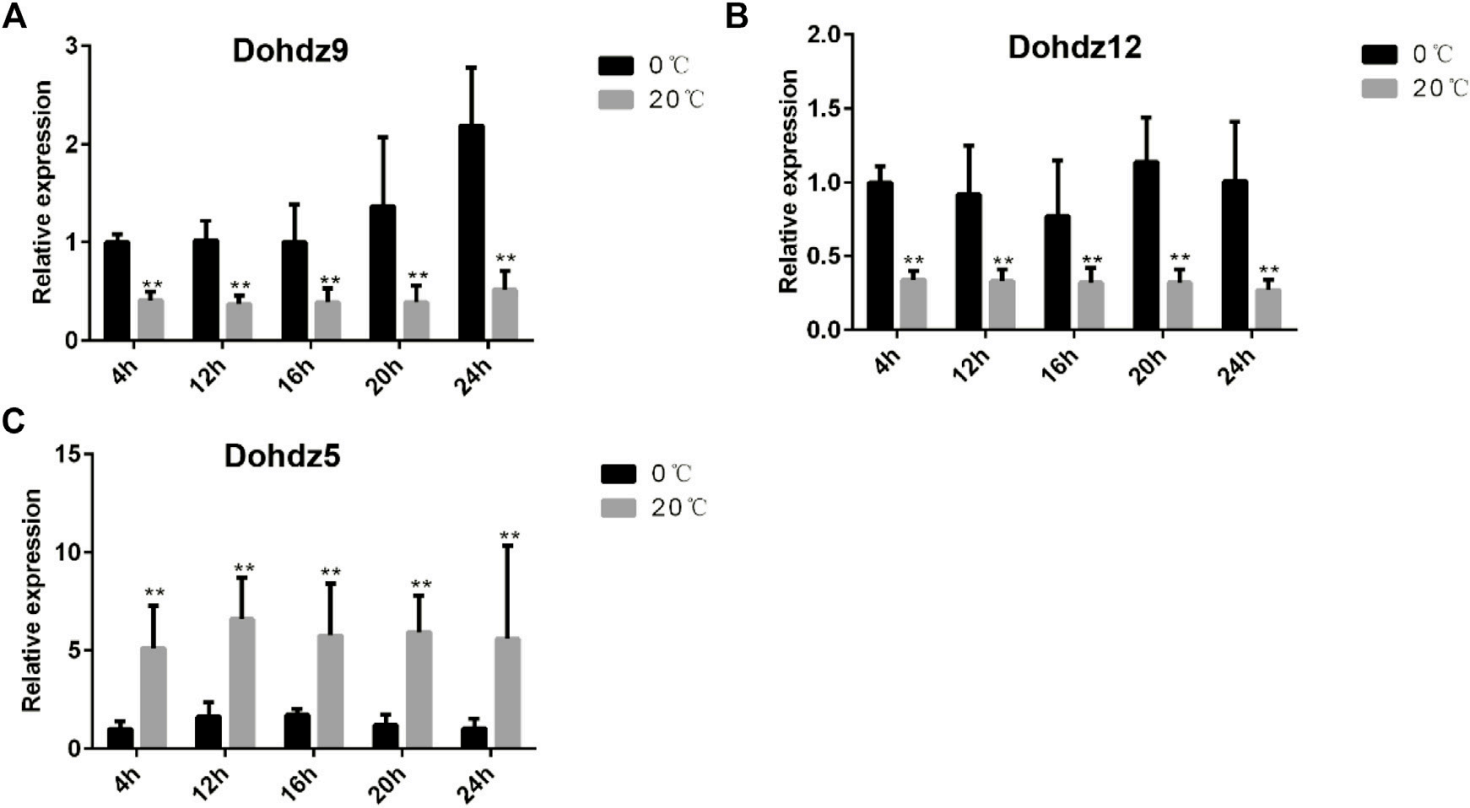
Expression patterns of three cold-responsive HD-ZIP genes under cold stress and normal conditions through qRT-PCR analysis. **(A–C)** expression patterns of Dohdz9, Dohdz12, and Dohdz5, respectively. **, *p* < 0.01 (Student’s t-test). Gene expression profiles were evaluated using the 2^−ΔΔCT^ methods.

To explore the key candidates associated with cold stress response, three cold-responsive Dohdzs (*Dohdz5*, downregulated; *Dohdz9;* and *Dohdz12*, upregulated) based on the RNA-seq analysis were selected to be verified by qRT-PCR analysis at 4, 12, 16, 20, and 24 h after chilling stress treatment (4°C) (Figure 6). The results found that the expression level of *Dohdz9* and *Dohdz12* was dramatically upregulated at all five-time courses under chilling stress compared to normal conditions, while *Dohdz5* was significantly downregulated at all timepoints, which was consistent with the RNA-seq analysis. These results demonstrated that *Dohdz5*, *Dohdz9*, and *Dohdz12* might be considered the key candidates underlying cold stress response, which provided the vital targets for further functional studies to reveal the molecular mechanism of HD-ZIP genes regulating cold adaptation and also help improve cold tolerance in *D. officinale* and beyond.

## 4 Discussion

*D. officinale* is an economically important Chinese herb with huge ornamental and medicinal values. However, the ornamental and medicinal values will be adversely affected when suffering from an unfavorable environment (Chew et al., 2013). Molecular regulation, physiological response, and signal transduction have been well documented to play crucial roles in abiotic stress tolerance in plants. The HD-ZIP family is one class of plant-specific transcription factors which functions as vital regulators to control diverse growth and developmental processes as well as in response to abiotic stresses in plants (Wang et al., 2013; Turchi et al., 2015). However, the significance of HD-ZIP genes is still unknown in *D. officinale.* Genome-wide identification of the given gene family not only provided valuable information about genomic organization, structure features, and evolutionary relationship of the members but also provided the candidates for further functional studies. In *Dendrobium officinale*, several gene families were also explored by genome-wide identification, such as MADS-box family genes, B-BOX genes, and WRKY family genes (He et al., 2017; Chen et al., 2019b; Cao et al., 2019). Therefore, we systematically investigated the HD-ZIP family in *Dendrobium officinale* at the genome-wide level in this study. Thirty-seven HD-ZIP genes were found in *D. officinale* through genome-wide search, which could be divided into four groups based on sequence homology, DNA binding specificity, and conserved motifs, namely, I, II, III, and IV subfamilies (Elhiti and Stasolla, 2009; Perotti et al., 2017). Furthermore, phylogenetic analysis supported the classification with 12, 16, two, and seven *Dohdz* genes belonging to the I to IV subfamilies, respectively. Previous studies have extensively demonstrated that HD-ZIP genes played crucial roles in regulating diverse developmental and physiological processes in plants (Wang et al., 2013). To get insights into the function of these Dohdz genes, we investigated the expression patterns of these genes in different tissues. We found that the members belonging to group I expressed in different tissues; group II mainly expressed in the stem, column, and sepal; group III and IV were tissue-specific and preferred to express in flower buds. These results demonstrated that different HD-ZIP genes contribute to the morphogenesis of different tissues. Several HD-ZIP genes from different groups also showed similar expression patterns. To know whether the conserved motifs are related to the expression profile of *Dohdz* genes, the protein motifs were further predicted using SMART software. A total of four conserved motifs were found among all the *Dohdz* genes, including HOX, START, HALZ, and BRLZ motifs. Members of HD-ZIP I and II contained the conserved HD and LZ domains, which could bind to the similar sequence CAAT-N-ATTG (Elhiti and Stasolla, 2009). HD-ZIP III and IV also contained a START domain with putative lipid-binding capability, except for the HD and LZ domains. Further analysis showed that HOX domains were found in all the Dohdz proteins, except for Dohdz19. Dohdz genes belonging to group II had only the HOX domain, while the members in group I and IV had several BRLZ and START motifs, respectively. Taken together, we speculated that the tissue-specific expression profile of *Dohdz* genes was significantly correlated with the conserved motifs. In addition, we found that the expression of HD-ZIP genes in *D. officinale* was more specific than that of HD-ZIP genes in *Arabidopsis* and rice, indicating that the HD-ZIP genes played more specific roles in participating in different processes associated with growth and development of *D. officinale.*

Several HD-ZIP genes have been revealed to participate in the cold stress response in plants. Kim et al. (2004) demonstrated that the expression of HD-ZIP genes was elevated under low temperature and drought stresses. Cabello et al. (2012b) showed that the homologous HD-ZIP I transcription factors, *HaHB1* and *AtHB13*, could confer cold tolerance *via* the induction of pathogenesis-related and glucanase proteins. Peng et al. (2015) and Li et al. (2017) found that HD-ZIP genes could regulate cold stress adaptation through transcriptomic profiling analysis in paper mulberry and maize. Recently, dynamic expression under abiotic stresses and phytohormone treatments indicated that most of *NtHD-ZIP IV* genes were induced by heat, cold, salt, and drought in *N. tabacum* (Zhang et al., 2019). In this study, 14 HD-ZIP genes were found to be significantly differentially expressed under cold stress. In addition, *Dohdz5*, *Dohdz9*, and *Dohdz12* showed the most significant changes in the expression when suffering cold stress, which was consistent with the fact that HD-ZIP genes in I and II subfamilies played important roles in abiotic stress tolerance. Furthermore, the expression patterns of *Dohdz5*, *Dohdz9*, and *Dohdz12* were validated through qRT-PCR analysis such that *Dohdz9* and *Dohdz12* showed dramatically upregulated expression upon chilling and then remained constant, while *Dohdz5* displayed downregulated expression upon chilling and remained constant thereafter. This result indicated that the HD-ZIP genes could act as both positive and negative regulators to participate in the chilling response process, which was consistent with the results in tomatoes (Zhang et al., 2014). Compared to *Dohdz9* and *Dohdz12*, *Dohdz5* possessed a specific ARE element in the promoter region, suggesting that *Dohdz5* might be involved in anerobic induction. The downregulated expression of *Dohdz5* could protect from chilling damage. These chilling-related genes provided vital candidates for further functional studies to improve cold tolerance in *D. officinale* and beyond.

## 5 Conclusion

This study systematically identified the HD-ZIP family in *D. officinale* through a genome-wide search method, and a total of 37 Dohdz genes were identified belonging to four subfamilies based on phylogenetic analysis and conserved motif compositions. Further analysis revealed that these HD-ZIP genes preferred tissue-specific expression, and their expression could be correlated with the conserved domains and *cis*-elements. Moreover, 14 *Dohdz* genes were differentially expressed under cold stress, of which *Dohdz5*, *Dohdz9*, and *Dohdz12* were considered to be key candidates for regulating cold stress response and tolerance. Overall, this study not only provided useful information on the genomic organization and evolutionary features of the HD-ZIP family but also provided important candidates for further functional study to better understand the molecular mechanism of HD-ZIP regulating cold stress response and tolerance in *D. officinale* and beyond*.*

## Data Availability

The raw data supporting the conclusion of this article will be made available by the authors, without undue reservation.

## Supplementary Material

Supplementary Figure 1 Tissue-specific expression analysis of HD-ZIP genes in *D. officinale*. Tau was calculated by Yanai et al. (2005). Tau varies from 0 to 1, where 0 means broadly expressed and 1 is specific.

Supplementary Figure 2 WGCNA analysis of the HD-ZIP genes and other genes based on the expression levels in nine different tissues and under cold stress in *D. officinale.*

## References

[B1] Agalou A. Purwantomo S. Overnäs E. Johannesson H. Zhu X. Estiati A. (2008). A Genome-wide Survey of HD-ZIP Genes in rice and Analysis of Drought-Responsive Family Members. Plant Mol. Biol. 66 (1-2), 87–103. 10.1007/s11103-007-9255-7 17999151

[B2] Ariel F. D. Manavella P. A. Dezar C. A. Chan R. L. (2007). The True story of the HD-ZIP Family. Trends Plant Science 12 (9), 419–426. 10.1016/j.tplants.2007.08.003 17698401

[B3] Cabello J. V. Arce A. L. Chan R. L. (2012). The Homologous HD-ZIP I Transcription Factors HaHB1 and AtHB13 Confer Cold Tolerance via the Induction of Pathogenesis-Related and Glucanase Proteins. Plant J. : Cel. Mol. Biol. 69 (1), 141–153. 10.1111/j.1365-313x.2011.04778.x 21899607

[B4] Cabello J. V. Arce A. L. Chan R. L. (2012). The Homologous HD-Zip I Transcription Factors HaHB1 and AtHB13 Confer Cold Tolerance via the Induction of Pathogenesis-Related and Glucanase proteinsHaHB1 and AtHB13 Confer Cold Tolerance via the Induction of Pathogenesis-Related and Glucanase Proteins. Plant J. 69, 141–153. 10.1111/j.1365-313x.2011.04778.x 21899607

[B5] Cao Y. Meng D. Han Y. Chen T. Jiao C. Chen Y. (2019). Comparative Analysis of B-BOX Genes and Their Expression Pattern Analysis under Various Treatments in Dendrobium Officinale. BMC Plant Biol. 19 (1), 245. 10.1186/s12870-019-1851-6 31182022 PMC6558717

[B6] Chen W. Cheng Z. Liu L. Wang M. You X. Wang J. (2019). Small Grain and Dwarf 2, Encoding an HD-ZIP II Family Transcription Factor, Regulates Plant Development by Modulating Gibberellin Biosynthesis in rice. Plant Sci. 288, 110208. 10.1016/j.plantsci.2019.110208 31521223

[B7] Chen Y. Shen Q. Lyu P. Lin R. Sun C. (2019). Identification and Expression Profiling of Selected MADS-Box Family Genes in Dendrobium Officinale. Genetica 147 (3-4), 303–313. 10.1007/s10709-019-00071-5 31292836

[B8] Chen Z. Yuan Y. Fu D. Shen C. Yang Y. (2017). Identification and Expression Profiling of the Auxin Response Factors in Dendrobium Officinale under Abiotic Stresses. Int. J. Mol. Sci. 18 (5). 10.3390/ijms18050927 PMC5454840 28471373

[B9] Chew W. Hrmova M. Lopato S. (2013). Role of Homeodomain Leucine Zipper (HD-ZIP) IV Transcription Factors in Plant Development and Plant protection from Deleterious Environmental Factors. Ijms 14 (4), 8122–8147. 10.3390/ijms14048122 23584027 PMC3645734

[B10] Cristina M. Sessa G. Dolan L. Linstead P. Baima S. Ruberti I. (1996). The Arabidopsis Athb-10 (GLABRA2) Is an HD-ZIP Protein Required for Regulation of Root Hair Development. Plant J. 10 (3), 393–402. 10.1046/j.1365-313x.1996.10030393.x 8811855

[B11] Damodaran S. Dubois A. Xie J. Ma Q. Hindié V. Subramanian S. (2019). GmZPR3d Interacts with GmHD-ZIP III Proteins and Regulates Soybean Root and Nodule Vascular Development. Int. J. Mol. Sci. 20 (4), 827. 10.3390/ijms20040827 PMC6412583 30769886

[B12] Elhiti M. Stasolla C. (2009). Structure and Function of Homodomain-Leucine Zipper (HD-ZIP) Proteins. Plant Signaling Behav. 4 (2), 86–88. 10.4161/psb.4.2.7692 PMC2637487 19649178

[B13] Gong S. Ding Y. Hu S. Ding L. Chen Z. Zhu C. (2019). The Role of HD‐Zip Class I Transcription Factors in Plant Response to Abiotic Stresses. Physiol. Plantarum 167 (4), 516–525. 10.1111/ppl.12965 30851063

[B14] Gu C. Guo Z.-H. Cheng H.-Y. Zhou Y.-H. Qi K.-J. Wang G.-M. (2019). A HD-ZIP II HOMEBOX Transcription Factor, PpHB.G7, Mediates Ethylene Biosynthesis during Fruit Ripening in Peach. Plant Sci. 278, 12–19. 10.1016/j.plantsci.2018.10.008 30471725

[B15] He C. Teixeira da Silva J. A. Tan J. Zhang J. Pan X. Li M. (2017). A Genome-wide Identification of the WRKY Family Genes and a Survey of Potential WRKY Target Genes in Dendrobium Officinale. Sci. Rep. 7 (1), 9200. 10.1038/s41598-017-07872-8 28835632 PMC5569039

[B16] He H. Lei Y. Yi Z. Raza A. Zeng L. Yan L. (2021). Study on the Mechanism of Exogenous Serotonin Improving Cold Tolerance of Rapeseed (Brassica Napus L.) Seedlings. Plant Growth Regul. 94, 161–170. 10.1007/s10725-021-00700-0

[B17] Henriksson E. Olsson A. S. B. Johannesson H. Johansson H. Hanson J. Engström P. (2005). Homeodomain Leucine Zipper Class I Genes in Arabidopsis. Expression Patterns and Phylogenetic Relationships. Plant Physiol. 139 (1), 509–518. 10.1104/pp.105.063461 16055682 PMC1203399

[B18] Hu R. Chi X. Chai G. Kong Y. He G. Wang X. (2012). Genome-wide Identification, Evolutionary Expansion, and Expression Profile of Homeodomain-Leucine Zipper Gene Family in poplar (Populus trichocarpa). PloS one 7 (2), e31149. 10.1371/journal.pone.0031149 22359569 PMC3281058

[B19] Hu W. Wang L. Tie W. Yan Y. Ding Z. Liu J. (2016). Genome-wide Analyses of the bZIP Family Reveal Their Involvement in the Development, Ripening and Abiotic Stress Response in Banana. Sci. Rep. 6, 30203. 10.1038/srep30203 27445085 PMC4957152

[B20] Kamata N. Okada H. Komeda Y. Takahashi T. (2013). Mutations in Epidermis-specific HD-ZIP IV Genes Affect floral Organ Identity inArabidopsis Thaliana. Plant J. 75 (3), 430–440. 10.1111/tpj.12211 23590515

[B21] Kim S. An C. S. Hong Y. N. Lee K. W. (2004). Cold-inducible Transcription Factor, CaCBF, Is Associated with a Homeodomain Leucine Zipper Protein in Hot Pepper (Capsicum Annuum L.). Mol. Cell 18 (3), 300–308. 10.14348/.1970.0.0 15650325

[B22] Kovalchuk N. Chew W. Sornaraj P. Borisjuk N. Yang N. Singh R. (2016). The Homeodomain Transcription Factor Ta HDZ ipI‐2 from Wheat Regulates Frost Tolerance, Flowering Time and Spike Development in Transgenic Barley. New Phytol. 211 (2), 671–687. 10.1111/nph.13919 26990681

[B23] Kovalchuk N. Wu W. Eini O. Bazanova N. Pallotta M. Shirley N. (2012). The Scutellar Vascular Bundle-specific Promoter of the Wheat HD-ZIP IV Transcription Factor Shows Similar Spatial and Temporal Activity in Transgenic Wheat, Barley and rice. Plant Biotechnol. J. 10 (1), 43–53. 10.1111/j.1467-7652.2011.00633.x 21689369

[B24] Li C. Ma X. Huang X. Wang H. Wu H. Zhao M. (2019). Involvement of HD-ZIP I Transcription Factors LcHB2 and LcHB3 in Fruitlet Abscission by Promoting Transcription of Genes Related to the Biosynthesis of Ethylene and ABA in Litchi. Tree Physiol. 39 (12), 1600–1613. 10.1093/treephys/tpz071 31222320

[B25] Li P. Cao W. Fang H. Xu S. Yin S. Zhang Y. (2017). Transcriptomic Profiling of the Maize (Zea mays L.) Leaf Response to Abiotic Stresses at the Seedling Stage. Front. Plant Sci. 8, 290. 10.3389/fpls.2017.00290 28298920 PMC5331654

[B26] Li W. Dong J. Cao M. Gao X. Wang D. Liu B. (2019). Genome-wide Identification and Characterization of HD-ZIP Genes in Potato. Gene 697, 103–117. 10.1016/j.gene.2019.02.024 30776460

[B27] Li Y. Bai B. Wen F. Zhao M. Xia Q. Yang D. H. (2019). Genome-Wide Identification and Expression Analysis of HD-ZIP I Gene Subfamily in Nicotiana Tabacum. Genes (Basel) 10 (8), 575. 10.3390/genes10080575 PMC6723700 31366162

[B28] Mao H. Yu L. Li Z. Liu H. Han R. (2016). Molecular Evolution and Gene Expression Differences within the HD-ZIP Transcription Factor Family of Zea mays L. Genetica 144 (2), 243–257. 10.1007/s10709-016-9896-z 26979310

[B29] Peng X. Wu Q. Teng L. Tang F. Pi Z. Shen S. (2015). Transcriptional Regulation of the Paper mulberry under Cold Stress as Revealed by a Comprehensive Analysis of Transcription Factors. BMC Plant Biol. 15, 108. 10.1186/s12870-015-0489-2 25928853 PMC4432934

[B30] Perotti M. F. Ribone P. A. Chan R. L. (2017). Plant Transcription Factors from the Homeodomain-Leucine Zipper Family I. Role in Development and Stress Responses. IUBMB life 69 (5), 280–289. 10.1002/iub.1619 28337836

[B31] Ponting C. P. Aravind L. (1999). START: a Lipid-Binding Domain in StAR, HD-ZIP and Signalling Proteins. Trends Biochemical Sciences 24 (4), 130–132. 10.1016/s0968-0004(99)01362-6 10322415

[B32] Ruberti I. Sessa G. Lucchetti S. Morelli G. (1991). A Novel Class of Plant Proteins Containing a Homeodomain with a Closely Linked Leucine Zipper Motif. EMBO J. 10 (7), 1787–1791. 10.1002/j.1460-2075.1991.tb07703.x 1675603 PMC452851

[B33] Sessa G. Carabelli M. Possenti M. Morelli G. Ruberti I. (2018). Multiple Links between HD-ZIP Proteins and Hormone Networks. Int. J. Mol. Sci. 19 (12), 4047. 10.3390/ijms19124047 PMC6320839 30558150

[B34] Sharif R. Raza A. Chen P. Li Y. El-Ballat E. M. Rauf A. (2021). HD-ZIP Gene Family: Potential Roles in Improving Plant Growth and Regulating Stress-Responsive Mechanisms in Plants. Genes 12 (8), 1256. 10.3390/genes12081256 34440430 PMC8394574

[B35] Turchi L. Baima S. Morelli G. Ruberti I. (2015). Interplay of HD-ZIP II and III Transcription Factors in Auxin-Regulated Plant Development. Exbotj 66 (16), 5043–5053. 10.1093/jxb/erv174 25911742

[B36] Vernoud V. Laigle G. Rozier F. Meeley R. B. Perez P. Rogowsky P. M. (2009). The HD-ZIP IV Transcription Factor OCL4 Is Necessary for Trichome Patterning and Anther Development in maize. Plant J. : Cel. Mol. Biol. 59 (6), 883–894. 10.1111/j.1365-313x.2009.03916.x 19453441

[B37] Wang H. Li G.-B. Zhang D.-Y. Lin J. Sheng B.-L. Han J.-L. (2013). Biological Functions of HD-Zip Transcription Factors. Hereditas (Beijing) 35 (10), 1179–1188. 10.3724/sp.j.1005.2013.01179 24459891

[B38] Wang Z. Wang S. Xiao Y. Li Z. Wu M. Xie X. (2020). Functional Characterization of a HD-ZIP IV Transcription Factor NtHDG2 in Regulating Flavonols Biosynthesis in Nicotiana Tabacum. Plant Physiol. Biochem. 146, 259–268. 10.1016/j.plaphy.2019.11.033 31778931

[B39] Yanai I. Benjamin H. Shmoish M. Chalifa-Caspi V. Shklar M. Ophir R. (2005). Genome-wide Midrange Transcription Profiles Reveal Expression Level Relationships in Human Tissue Specification. Bioinformatics 21 (5), 650–659. 10.1093/bioinformatics/bti042 15388519

[B40] Yang Y. Luang S. Harris J. Riboni M. Li Y. Bazanova N. (2018). Overexpression of the Class I Homeodomain Transcription Factor TaHDZipI-5 Increases Drought and Frost Tolerance in Transgenic Wheat. Plant Biotechnol. J. 16 (6), 1227–1240. 10.1111/pbi.12865 29193733 PMC5978581

[B41] Yang Y. Luang S. Harris J. Riboni M. Li Y. Bazanova N. (2018). Overexpression of the Class I Homeodomain Transcription Factor TaHDZipI-5 Increases Drought and Frost Tolerance in Transgenic wheatTaHDZipI-5 Increases Drought and Frost Tolerance in Transgenic Wheat. Plant Biotechnol. J. 16, 1227–1240. 10.1111/pbi.12865 29193733 PMC5978581

[B42] Yuan Y. Zhang J. Liu X. Meng M. Wang J. Lin J. (2020). Tissue-specific Transcriptome for Dendrobium Officinale Reveals Genes Involved in Flavonoid Biosynthesis. Genomics 112 (2), 1781–1794. 10.1016/j.ygeno.2019.10.010 31678153

[B43] Zhang H. Ma X. Li W. Niu D. Wang Z. Yan X. (2019). Genome-wide Characterization of NtHD-ZIP IV: Different Roles in Abiotic Stress Response and Glandular Trichome Induction. BMC Plant Biol. 19 (1), 444. 10.1186/s12870-019-2023-4 31651252 PMC6814048

[B44] Zhang Z. Chen X. Guan X. Liu Y. Chen H. Wang T. (2014). A Genome-wide Survey of Homeodomain-Leucine Zipper Genes and Analysis of Cold-Responsive HD-Zip I Members' Expression in Tomato. Biosci. Biotechnol. Biochem. 78 (8), 1337–1349. 10.1080/09168451.2014.923292 25130735

[B45] Zhao Y. Zhou Y. Jiang H. Li X. Gan D. Peng X. (2011). Systematic Analysis of Sequences and Expression Patterns of Drought-Responsive Members of the HD-ZIP Gene Family in maize. PloS one 6 (12), e28488. 10.1371/journal.pone.0028488 22164299 PMC3229603

