# Supplementary figures and images for: Genome-Wide Characterization and Expression Analysis of HD-ZIP Gene Family in Dendrobium officinale
Source: Front Genet. 2022 Mar 18;13:797014. doi: 10.3389/fgene.2022.797014 (PMC8971680; doi:10.3389/fgene.2022.797014)

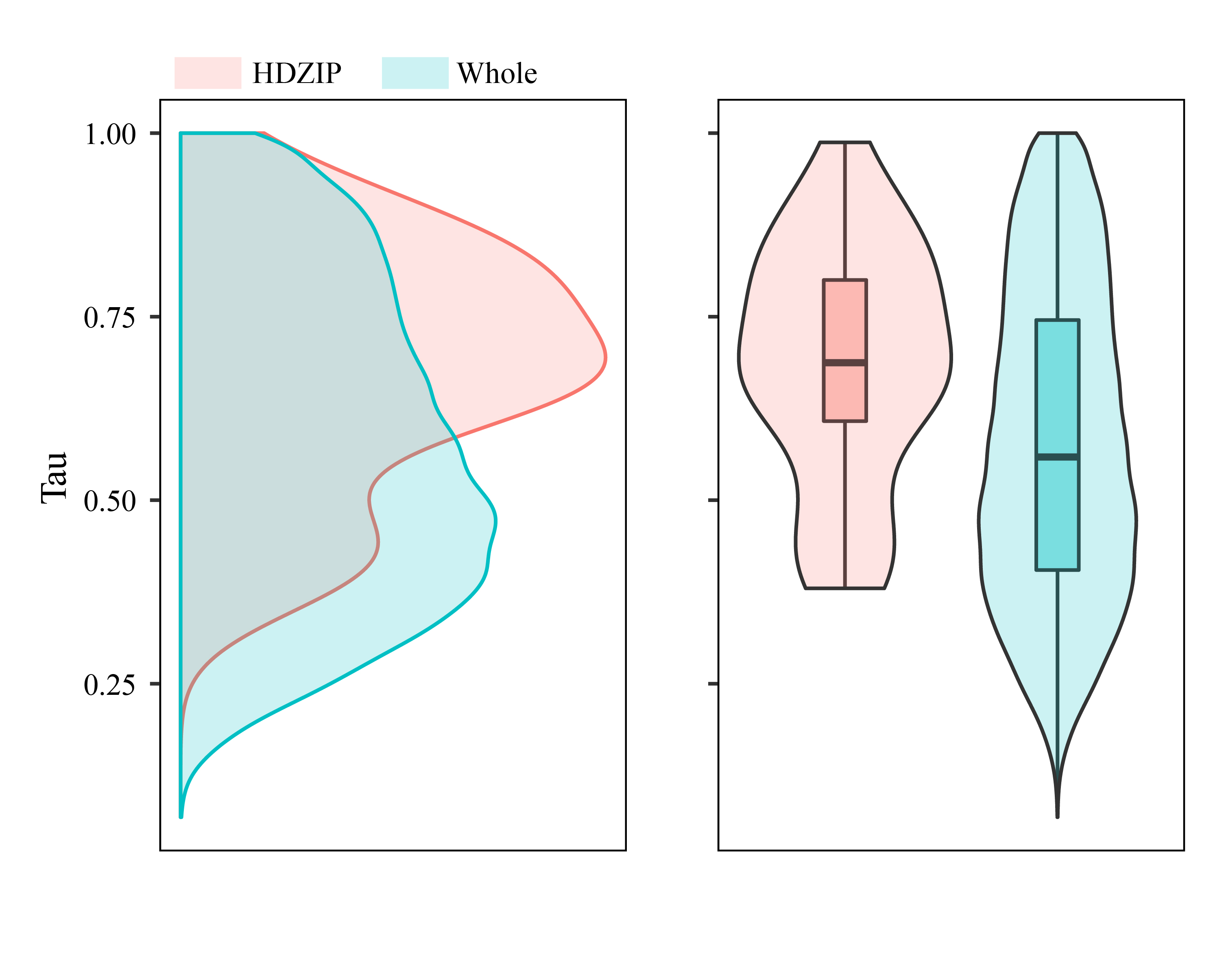

Supplement: Supplementary file 1 [file Image1.JPEG]
